# Educating the Public Health Workforce

**DOI:** 10.3389/fpubh.2013.00020

**Published:** 2013-07-18

**Authors:** Connie Joann Evashwick

**Affiliations:** ^1^College of Health and Human Services, George Mason University, Fairfax, VA, USA

The section *“Public Health Education and Promotion”* of the journal *“Frontiers in Public Health”* is the first initiative to focus specifically upon the pedagogy pertaining to the current and future public health workforce. In *Health Professionals for a New Century*, an international task force chaired by Harvard School of Public Health Dean Julio Frenk and Chinese Medical Board President Lincoln Chen examined education worldwide for medicine, nursing, and public health (1). They concluded that the field of public health has comparatively little literature about what, why, and how to teach. The implications challenge those educating the public health workforce to prove that we *do* think about what we teach, why we teach it, and how to teach effectively.

*Public Health Education and Promotion* is a single section with two distinct components, bridged by the shared themes of (1) education of lay and professionals for effective public health practice, and (2) a need for a new vision to shape future changes. The mission of the Education component is to be a forum for discussions about the pedagogy relevant to the public health workforce. For shorthand, education to train the current and future public health workforce will be referred to as education *for* public health. The Promotion component will entertain articles pertaining to education *about* public health and new paradigms to *promote* public health at individual *and* community levels.

Educating the public health workforce is a critical and timely issue. A workforce strong in both capability and capacity is essential to the functioning of the public health system (2). And, as with so many other contemporary issues, the world has gone global, and public health is global (3). International emergencies, from the spread of infectious diseases such as SARS and avian flu, to natural and manmade disasters, such as the tsunami in Japan and the resulting degeneration of its nuclear power facilities, leave little doubt that public health must be addressed at a global level. This then necessitates a world-wide workforce of well-trained public health professionals capable of working in concert across disciplines, customs, and languages. A well-educated public health workforce is one of the most notable challenges to achieving a healthy world. However, the World Health Organization, as well as many individual nations, project severe shortages in the public health workforce (4).

The teaching of public health is more complicated than that of a single clinical discipline because public health is at once a discrete field and one that intersects with numerous others. Because public health is global and interdisciplinary, discussions about pedagogy must similarly engage representatives of multiple disciplines and geographic areas. The ultimate goal, of course, is to enhance the education of the current and future public health workforce to be effective in practicing public health around the globe.

Trends in public health education enhance the significance of a world-wide discussion about pedagogy. The number of schools of public health has expanded markedly in recent years. India offers a dramatic example. Having had graduate training programs in public health through medical and health sciences schools for many years, India declared the need for distinct schools of public health. In 2006, India launched an initiative led by the Public Health Foundation of India to start five to seven new institutes of public health, as well as strengthen existing training programs and increase research capacity (5). In the United States (US), the number of accredited schools of public health expanded from 24 in 1988 to 50 by the end of 2012 (6, 7). Public health as an undergraduate major is burgeoning, from Australia to global on-line programs such as offered by Walden University (8, 9). The US-based Council on Education for Public Health will soon begin to offer accreditation for undergraduate programs (10). DrPH programs are springing up from Texas to the United Arab Emirates. This growth is impressive – but it also increases the need for communication and exchange about the education being offered. For the field to develop world-wide standards, common expectations of outcomes, criteria for cross-national recognition of educational credentials, and interprofessional engagement, serious attention to the underlying pedagogy is warranted. An on-line, open access, rapid publication journal is one way to facilitate such communication.

A review of the literature pertaining to the pedagogy of education for public health is currently underway. An initial search of literature recorded in three major data bases produced nearly 700 citations for the period 2000–2012. Article topics cover a wide range of subjects, including education targeted at undergraduate, master's level, doctoral, and workforce audiences; international and global health; interprofessional education, with public health as itself an integrating discipline; competencies, curricula, evaluation, and innovative teaching techniques; cross-cultural competencies; and intersection of the public health workforce with the healthcare delivery workforce.

At the same time that public health seeks to build its pedagogy, the field of education is evolving at the macro level. Competencies have replaced courses and curricula as the major forces shaping education. The methods of teaching are also changing as technology evolves and the younger generations want new methods to learn and demonstrate performance. In the field of public health, recognition is emerging of changes in education related to health promotion, from changing behavior of individuals to education aimed at changing the actions of communities. Not only must we educate the future public health workforce, we must help those already working in public health to stay current, think globally, and engage with new technology. We must train those working in the field how to teach others about public health with techniques that are evolving and content that is continuously changing.

Clearly, educating the public health workforce is a complicated task, with many moving parts. Compiling evidence for what we know about effective teaching can facilitate better teaching, achieve more profound learning, and provide the assurance that those teaching others have the tools and the motivation to continue to advance the pedagogy as well as content of public health.

## Obstacles to Producing Pedagogy for Public Health

Several obstacles confront those seeking to understand the pedagogy for public health. The issues outlined below are expected to be addressed by authors worldwide through articles based on research, literature reviews, descriptions of innovative programs, evaluation results, personal experience, and reasoned opinion. *Public Health Education and Promotion* will provide a place for publications but also blogs for active discussion, editorials, and commentaries to provoke thinking.

### Public health jobs vary greatly

Public health covers a broad spectrum of activities, ranging from vaccination programs in rural Africa to educational programs for teens about birth control in developed countries to commercial enterprises “going green.” Whether there is a single common educational track appropriate for all is a subject for debate. The World Health Organization definition of basic public health functions might be used as core competencies – but the United States Centers for Disease Control and Prevention has a different definition of public health “essentials,” and other nations, too, define public health education outcomes related to their own needs (11). Which framework, with what set of outcomes, among the many that have been put forward, should underpin the pedagogy? Commonalities, differences, and underlying rationale for selecting an educational framework are all subjects worthy of analysis.

### Finding the literature

Most health professions have at least one journal with a specific focus on the education of the profession. With no existing journal clearly identified as dedicated to the education of the public health workforce, peer review published articles are scattered in various journals or lay buried in the “gray” literature, often as a government or private sector report. A review of articles reported in PubMed between 2000 and 2012 revealed 490 articles published in over 150 journals. A simple search of “public health education” yields thousands of articles ranging from messages about using condoms to the newest techniques to promote smoking cessation. Public health needs a sophisticated Boolean search pattern to find relevant articles, and even then, many articles are buried in journals of public health practice or specific disciplines, with resulting Key Words that do not lend to easy identification. When articles are hard to find, their wisdom is not shared as widely as possible. *Public Health Education and Promotion* strives to address this problem by providing a focus on pedagogy, organizing special issues, and offering guidance on how to cite key works and conduct productive searches. We have engaged a medical librarian to help explain optimum search techniques, and an article explaining how to search for “education for public health” will be one of the journal's first contributions to the field.

### Risk of writing

The lack of a journal has two other disadvantages: it leads to the assumption that those in public health do not think about what they teach or how they teach it, and, it discourages anyone who might want to publish from bothering to write up their research, evaluation, or thoughts, as there is no obvious journal to target. Any person who has ever been on a tenured faculty track knows that a key consideration in selecting what to study and write about is the likelihood that the work can get published. Articles are shaped to fit the desired journal's target audience. This inhibits educators in public health from sharing with each other new approaches to education or evaluation revealing the effectiveness of various teaching techniques. Being created by a network of scientists, *Public Health Education and Promotion* provides a peer-oriented, author-friendly venue to submit articles, with an 80% acceptance rate, and a mentoring system that links reviewers with authors in order to streamline resubmission and make changes efficiently yet with the ultimate outcome of high quality.

### Interdisciplinary nature

Public health is arguably an independent discipline (12). In the United States, as in Great Britain and other countries, public health began as a specialization within medicine, nursing, environmental engineering, laboratory science, among others. And, despite growing recognition of public health as a distinct field, it remains a specialty within clinical, environmental, administrative, and legal disciplines. This requires that discussions about the pedagogy of public health be intertwined with discussions of the pedagogy of other fields. This very complexity makes it all the more essential that educators share their thinking and their experiences. Meanwhile, interprofessional education among the health professions is a high profile topic at the moment (13), and public health is a perfect place to share the literature on effective methods of teaching and learning interprofessional behavior. The Associate Editors have a wide range of backgrounds, which will help the journal reach out to and appreciate authors from all health professions disciplines.

### Competency revolution

As the world of education has moved in the direction of competencies as the underpinning for curricula, numerous sets of competencies for the health field have emerged, including interprofessional competencies, cultural competencies, undergraduate competencies, graduate competencies, workforce competencies – with such distinctions repeated by over and over again by different countries (India, Australia, United States, New Zealand, among numerous others). Public health pedagogy must accept, adopt, adapt, reject, intersperse – but at minimum consider each of these and guide individual educators, institutions, and nations in understanding what is core and what is supplemental. As a community of educators, we need to help each other with how to craft education relevant for a particular organization, country, and target audience.

### Education, accreditation, and employment are not aligned

In contrast to clinical disciplines such as medicine, public health has only recently begun the path toward standardization of education (competencies designed by educators and practitioners), accreditation (of educational institutions), professional certification (of individuals based on objective criteria established by the field), and licensure/regulation (based on criteria legally mandated by government). However, even this varies across nations. Under the Bologna Declaration of 2000, European and four additional nations committed to standardization in workforce certification (14). The United States has taken a different approach, and recently implemented a certification exam for individuals having MPH or equivalent academic degrees (15). As a result of this lack of alignment, the world-wide workforce cannot be counted or analyzed with certainty based on education. To address public health problems globally and foster cross-national cooperation, understanding of public health credentials is essential. *Public Health Education and Promotion* strives to aid in understanding through its international focus on pedagogy and through planned special issues on the status of pubic health education in different countries and regions.

## Conclusion

Education for professionals working in public health is evolving in exciting and complex ways. Those who are involved in the field have the opportunity to shape education worldwide, for the ultimate benefit of creating healthy populations served by well trained professionals with a broad understanding of the forces that impact the health of individuals and communities. *Public Health Education and Promotion* will lead the international dialog and be on the forefront of addressing complicated education and workforce issues with the expectation not of a singular outcome, but of a rich discussion.
